# *COL1A1* and *FGFR2* Single-Nucleotide Polymorphisms Found in Class II and Class III Skeletal Malocclusions in Javanese Population

**DOI:** 10.1055/s-0042-1744371

**Published:** 2022-06-07

**Authors:** I Gusti Aju Wahju Ardani, Melisa Budipramana, Erlina Rachmawati, Alexander Patera Nugraha, I Kade Karisma Gita Ardana, Theresia Indah Budhy, Rozita Hassan, Dwi Listyorini, Riyanarto Sarno

**Affiliations:** 1 Department of Orthodontics, Faculty of Dental Medicine, Universitas Airlangga, Surabaya, Indonesia; 2 Department of Orthodontics, Faculty of Dental Medicine, Universitas Lambung Mangkurat, Banjarmasin, Indonesia; 3 Department of Biology, Faculty of Mathematics and Natural Sciences, Universitas Negeri Malang, Malang, Indonesia; 4 Department of Oral and Maxillofacial Pathology, Faculty of Dental Medicine, Universitas Airlangga, Surabaya, Indonesia; 5 Orthodontic Unit, School of Dental Sciences, Universiti Sains Malaysia, Kelantan, Malaysia; 6 Department of Biotechnology, Faculty of Mathematics and Natural Sciences, Universitas Negeri Malang, Malang, Indonesia; 7 Department of Informatics, Institute Technology of Sepuluh Nopember, Surabaya, Indonesia

**Keywords:** *COL1A1*, *FGFR2*, single-nucleotide polymorphisms, skeletal malocclusion, medicine

## Abstract

**Objective** The aim of this article is to analyze and compare the presence of single-nucleotide polymorphisms (SNPs) of *COL1A1* and *FGFR2* in class II and class III Javanese populations.

**Materials and Methods** Cephalometric radiographs from total 63 patients of class II and III were analyzed. SNP analysis was performed based on both *COL1A1* and *FGFR2* sequences amplified from total DNA of patients' fresh blood. Principal component analysis was done to calculate the data and find the correlation of the cephalometric indicators influenced by each mutation. *t* -test and Mann–Whitney analysis were performed to check the significance of differences occurred in each studied parameter ( *p* < 0.05).

**Result** There were three *COL1A1* SNPs found in class II and only two in class III, while three *FGFR2* SNPs found in both classes. Class II phenotype seemed to be strongly influenced by Y-axis and mandibular plane angle, while class III by lower gonial angle and mandibular plane angle.

**Conclusion** Based on this study, we suggest that rs2249492 of *COL1A1* and rs2981582 of *FGFR2* play important roles in class III, while rs2277632 of *COL1A1* and rs2981582 of *FGFR2* play important role in class II skeletal malocclusion in Javanese population.

## Introduction

Malocclusion is disharmony of dental and craniofacial growth and development. It can cause disruption of facial esthetics, inability to produce correct articulation, and chewing problem. In addition, there is an increased susceptibility to dental trauma and periodontal disease. 1 Angle classified malocclusion based on the anteroposterior position of the maxilla to the mandible, namely class I, II, and III malocclusion. 2 Malocclusion is caused by genetic and environmental influences. The fact that a certain type of malocclusion tends to be inherited in a family confirms that malocclusion is strongly influenced by genetic. 3 This causes the prevalence of malocclusion to vary among population groups. The prevalence of class II malocclusion is 22.9% among Caucasians, while it is only 5.1% in African population. 4 Class III malocclusion prevalence in Asian is 8 to 40%, while in African it is 3 to 8%. Its prevalence is lower in European, which is 0.48 to 4%. 5 The prevalence of class II malocclusion in Faculty of Dental Medicine, Airlangga University Dental Hospital in 2017 to 2018 is 27.04%, and class III is 19.74% from 233 patients. 6

Gene polymorphism is the presence of two or more genotype variations that appear in a certain frequency in a population. One of the most common form of gene polymorphism is single-nucleotide polymorphisms (SNPs). SNPs are mutation in one nucleotide, which can change the amino acid composition. These amino acids will have differentiated into the bone or muscles that establish variations in malocclusions. SNPs can occur in promoter genes, exons, and introns. SNPs can greatly affect gene expression through various mechanisms, including changing promoter activity, binding with transcription factors, causing DNA methylation and histone modification, and altering gene transcription and translation. In addition, amino acid substitution caused by the presence of SNPs can change amino acid sequence, secondary protein structure, protein function, and its interactions. 7

There are several genes associated with mandibular development that can affect skeletal malocclusion phenotype, for example, *PRRX1* , *COL11A1* , *SATB2* , *BMP3, MSX, EDN1, RUNX2, PAX5, DLX3, TBX1, COL1A1* , and *FGFR2* . The *COL1A1* and *FGFR2* genes were selected in this study because their SNPs' odd ratio values were greatest in class III skeletal malocclusion. 8 Mutations in *COL1A1* have been found in patients with osteogenesis imperfecta (OMIM 166200) and Ehlers-Danlos syndrome (OMIM 130000). Facial anomalies, including micrognathia, frontal bossing, and midface hypoplasia, are commonly found in both diseases. 8 Mutations in *FGFR2* cause Crouzon (OMIM 123500) and Apert syndrome (OMIM 101200). Both diseases have class III malocclusion. 9 However, the presence of SNPs in these genes in other population groups is still unknown.

Based on these facts, it is necessary to carry out case–control study to determine the SNPs in the *COL1A1* and *FGFR2* genes among Javanese population in Surabaya with class II and class III skeletal malocclusion phenotype. We assumed that this study will find SNPs *COL1A1* and *FGFR2* in class II and class III malocclusion. Furthermore, the aim of this study is to analyze the presence of SNPs in *COL1A1* and *FGFR2* in class II and class III Javanese skeletal malocclusion patients.

## Materials and Methods

### Ethical Statement

This ethical clearance was approved by number 042/HRECC.FODM/II/2020; all these samples are willing to follow this study with fulfill written inform consent.

### Sample Size

During the research time frame, there were 494 (78.5%), 69 (11%) and 66 (10.5%) patients with class I, II, and III skeletal malocclusions, respectively.

### Inclusion and Exclusion Criteria

The inclusion criteria were Javanese individuals, ANB ≥ 4 degrees and large overjet (for class II malocclusion) or ANB ≤ 0 degrees and negative overjet (for class III malocclusion), and minimum age of 18 years old. Any patient with previous history of orthodontic treatment or orthognathic surgery, craniofacial deformity or genetic syndrome, missing teeth, and supernumerary teeth is excluded from this study.

### Cephalometric Analysis

**Fig. 1:**
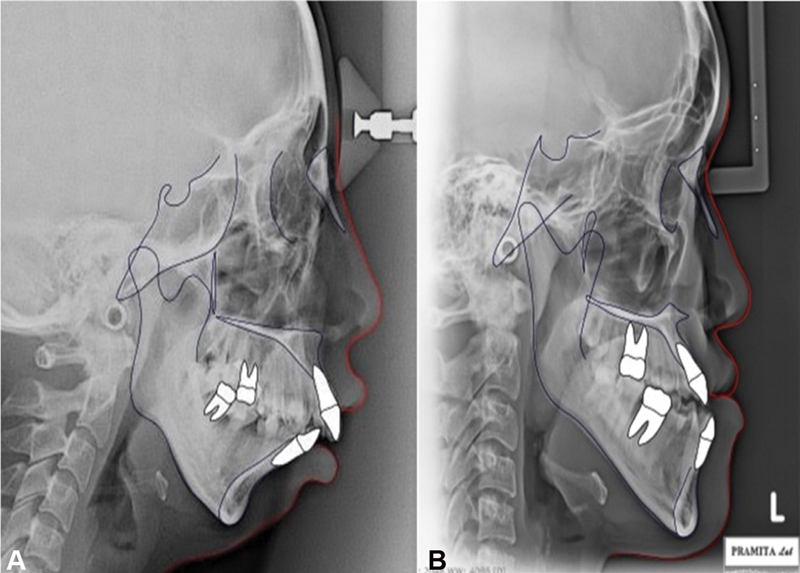
Cephalometric tracing of ( **A** ) class II and ( **B** ) class III skeletal malocclusion.

Only 31 class II and 32 class III skeletal malocclusion patients met the criteria of this study. The examples of cephalometric images are shown in Fig. 1 . Their pretreatment cephalometric radiographs were analyzed based on the various cephalometry measurements using OrthoVision (Vatech, South Korea) by two examiners. These various cephalometry measurements are as follows: saddle angle is formed from meeting point nasion (N)-sella (S)-articular (Ar), while articular angle is formed from meeting point of sella (S)-articular (Ar)-gonion (Go). Facial axis is angle formed from the meeting nasion(N)-basion (Ba) line and pterygoid(Pt)-Gnathion (Gn) line. Facial angle is the angle formed from the meeting Porion (P)-orbital (O) line with nasion (N)-pogonion (Pog) line. ANB is an angle formed from the meeting point maxillary deepest point (A)-nasion (N)-mandibular deepest point (B). Y-axis is the angle that formed from intersection of the lines P-O and the S-Gn line. Mandibular plane angle is the angle formed by the intersection of the lines P-O and Go-Gn. Upper gonial angle is angle formed by Ar-Go-N meeting point. Lower gonial angle is angle formed by meeting point N-Go-Me. Occlusal plane angle is angle formed by occlusal plane line meeting and the P-O line. Mandibular maxilla length difference is the difference mandibular linear length (condyle [Co]-Gn) with linear length maxilla (condyle [Co]-A). Posterior cranial base ratio and mandibular height are cranial base linear length ratio posterior (S-Ar) with height mandible (Ar-Go). The position of the ramus is an angle formed from the meeting P-O and CX-Go lines. Anterior face height ratio is the ratio of face height lower anterior (ANS-Me) and total anterior facial height (N-Me). Posterior face height ratio is the ratio of face height posterior (S-Go) and high total anterior face (N-Me).

### SNP Analysis

DNA was isolated from patient's fresh blood samples (QIAamp DNA Blood Mini Kit, Qiagen Inc., Basel, Switzerland) followed with polymerase chain reaction (PCR) based on *COL1A1* forward primer: 5′-CGTGACCTCAAGATGTGCCA-3′; and reverse primer: 5′- TTACAGGAAGCAGACAGGGC-3′. The primer used in *FGFR2* are: 5′-TGCCATCCCTCTGTGTAACG-3′ (forward primer); 5′-GTGGCTGCACGTATCTGAGT-3′ (reverse primer). Then, PCR products were undergone electrophoresis using 1% agarose in Tris-borat EDTA (TBE). Electrophoresis was performed with a voltage of 50 V in 1 hour. Samples were sequenced using the Sanger sequencing method. The sequencing results were then translated in Bioedit application and the translation results were aligned with the amino acid arrangement of each domain downloaded from National Center for Biotechnology Information (NCBI) Web site.

### Statistical Analysis

Sequences obtained than aligned using Clustal W to place the mutation. Then, this data will be recapitulated by Statistical Package for Social Science (SPSS) 17 version (IBM Corporation, Chicago, Illinois, United States). Principal component analysis (PCA) was done to calculate the data and find the correlation of the cephalometric indicators influenced by each mutation. Then, odds ratio, *t* -test, and also Mann–Whitney U test were conducted as supporting data. *t* -test and Mann-Whitney U test ( *p* < 0.05) are chosen by Shapiro–Wilk data homogeneity test and Levene's data normality test ( *p* > 0.05).

## Results

Cranial base cephalometric measurements (saddle angle and articular angle) are in normal range in both class II and class III skeletal malocclusion ( Fig. 1 ). Characteristics of class II skeletal are large overjet, convex profile, and mandibula rotation counterclockwise ( Fig. 1 ). The other side, characteristics of class III skeletal are crossbite anterior, concave profile, and mandibula growth forward ( Fig. 1 ). However, anteroposterior measurements, such as facial axis and ANB, show contradictive values as class II and class III malocclusions are categorized according to anteroposterior jaw relationship. Vertical measurements, such as facial angle, Y-axis, Frankfurt mandibular plane, gonial angle, occlusal plane angle, and facial height ratio, show variable results. The value of mandibular–maxillary length difference and ramus position are above and below the normal range, respectively. The length difference in class III is higher than that in class II, while ramus position in class II is lower than that in class III ( Table 1 ).

**Table 1:** Descriptive statistics of cephalometric measurements in class II and class III skeletal malocclusion

Cephalometric measurements	Class II	Class III	Normal range
Saddle angle	126.65 ± 7.59	124.20 ± 5.51	125.90 ± 4.40
Articular angle	149.97 ± 11.62	146.93 ± 6.80	148.70 ± 5.70
Facial axis	−10.86 ± 5.83	0.38 ± 4.72	-3.65 ± 3.58
ANB	7.20 ± 2.07	−3.97 ± 2.65	2.40 ± 1.80
Facial angle	78.58 ± 5.94	88.94 ± 4.57	89.00 ± 2.60
Y-axis	71.14 ± 6.82	62.53 ± 5.75	61.00 ± 2.80
Frankfurt mandibular plane	40.10 ± 10.24	29.36 ± 8.31	24.20 ± 4.60
Upper gonial angle	47.68 ± 11.04	46.68 ± 3.42	53.50 ± 1.50
Lower gonial angle	82.03 ± 7.01	76.20 ± 6.68	72.50 ± 2.50
Occlusal plane angle	22.08 ± 6.81	16.67 ± 4.48	17.92 ± 3.78
Mandibular-maxillary length difference	31.20 ± 10.00	47.91 ± 6.17	28.00 ± 3.20
Posterior cranial base/mandibular height ratio	0.85 ± 0.21	0.85 ± 0.25	0.72 ± 0.08
Ramus position	56.84 ± 7.56	62.78 ± 4.75	73.60 ± 3.50
Anterior facial height ratio	56.37 ± 2.94	55.02 ± 2.61	55.45 ± 1.79
Posterior facial height ratio	59.01 ± 6.54	65.12 ± 4.70	66.82 ± 4.25

Independent *t* -test and Mann–Whitney analysis revealed that all cephalometric indicators show significant difference between class II and class III malocclusion except cranial base measurements (saddle angle and articular angle), upper gonial angle, posterior cranial base/mandibular height ratio, and anterior facial height ratio ( Table 2 ).

**Table 2:** Significant difference of mean between class II and class III measurements

Cephalometric measurements	Significance	
	*t* -Test	Mann–Whitney U test
Saddle angle	0.148	
Articular angle	0.208	
Facial axis	0.000 a	
ANB	0.000 a	
Facial angle	0.000 a	
Y-axis	0.000 a	
Frankfurt mandibular plane	0.000 a	
Upper gonial angle		0.257
Lower gonial angle	0.001 a	
Occlusal plane angle		0.001 a
Mandibular-maxillary length difference	0.000 a	
Posterior cranial base/mandibular height ratio	0.940	
Ramus position	0.000 a	
Anterior facial height ratio	0.058	
Posterior facial height ratio	0.000 a	

a Significant difference ( *p* < 0.05).

**Fig. 2:**
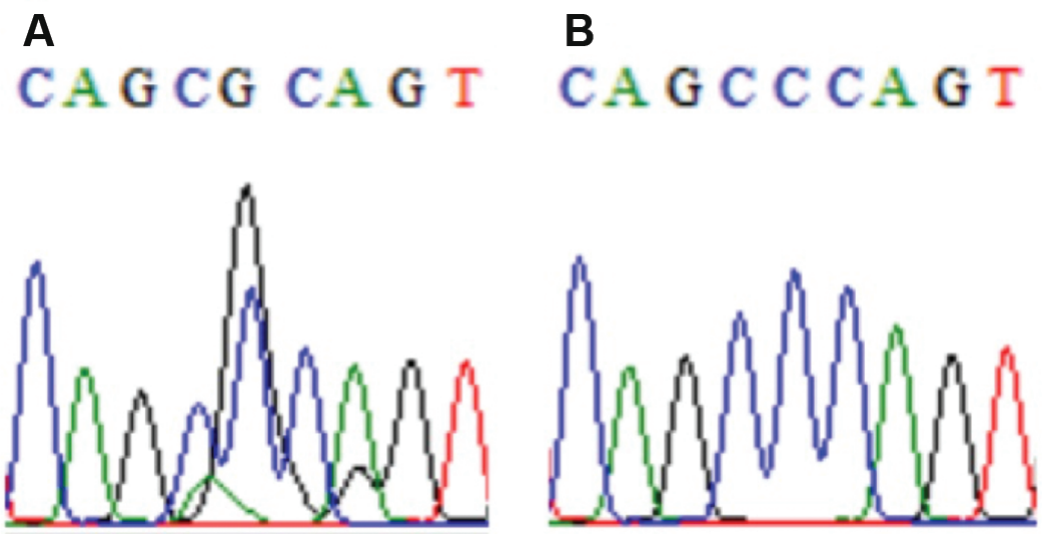
Mal II/COL1A1 heterozygote 286C/G in sample no. 20 ( **A** ). Sample no 19 ( **B** ) exhibits a homozygosity.

**Fig. 3:**
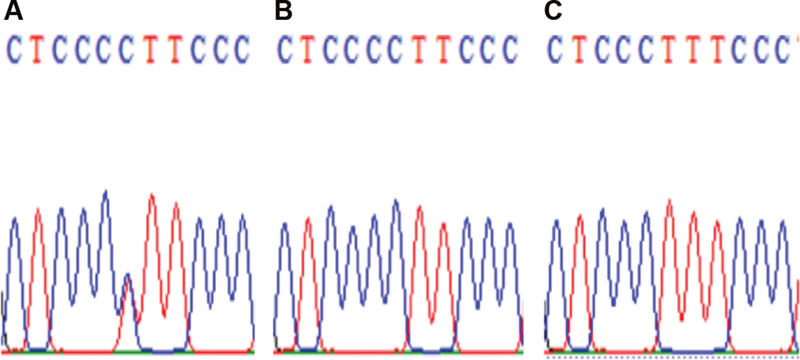
T/C153 heterozygosity and T153C substitution in Mal II/COL1A1. ( **A** ) Sample no. 19; ( **B** ) sample no. 24; (C) sample no. 25.

**Fig. 4:**
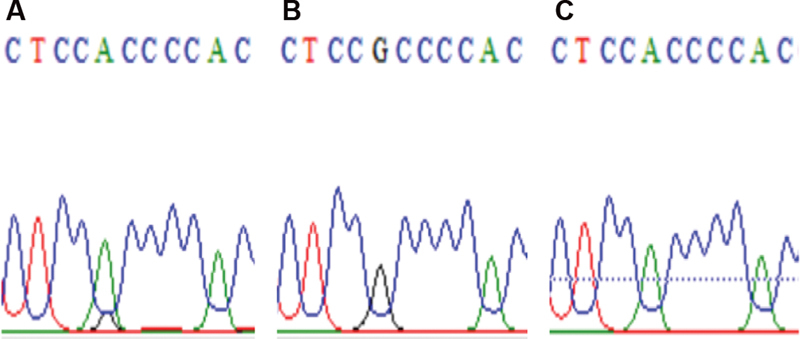
Intron G/A heterozygosity and G/A substitution in Mal III/COL1A1. ( **A** ) G/A heterozygote sample; ( **B** ) normal sample; ( **C** ) G/A substitutive mutation. Examples from samples no. 31, 41, and 49, respectively.

**Fig. 5:**
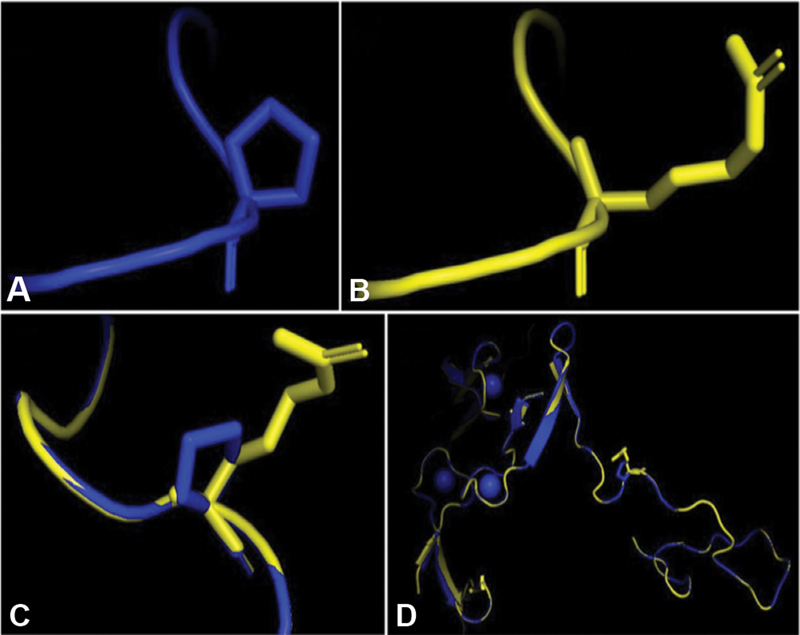
Protein structure changing due to the mutation. ( **A** ) Normal control; ( **B** ) mutant (sample no. 20); ( **C** ) normal-mutant alignment; and ( **D** ) whole sequence alignment showing the position of the mutation (white circle). Normal sequence in blue, mutant sequence drawn in yellow.

Mal II/COL1A1 heterozygote 286C/G in sample no. 20, no 19 exhibits a homozygosity ( Fig. 2 ). T/C153 heterozygosity and T153C substitution in Mal II/COL1A1 in sample no. 19, sample no. 24, and sample no. 25 ( Fig. 3 ). Intron G/A heterozygosity and G/A substitution in Mal III/COL1A1. Similarly to samples 31, 41, and 49 substitution in G/A, respectively.( Fig. 4 ). Protein structure changes due to the mutation that is found in sample no. 20. The whole sequence alignment showing the position of the mutation shown in Fig. 5 .

In *COL1A1* , there are three SNPs found in a significant number in class II samples: rs2277632T/C (65.1%), rs2249492G/A (49.6%), and rs50186619C/T (24.8). In class III skeletal malocclusion, there are only two SNPs found: rs2249492G/A (75%) and rs2277632T/C (46.9%). There are three significant SNPs in *FGFR2* in both class II and class III malocclusion: rs2981582T/C (83.7% in class II, 78.1% in class III), rs2162540G/A (74.4% in class II, 68.75% in class III), and rs3135724G/A (31% in class II, 46.9% in class III) ( Table 3 ).

**Table 3:** SNPs found in *COL1A1* and *FGFR2*

Gene	SNPs	Mutation	Class II	Class III
*COL1A1*	rs2277632	T/C	65.1%	46.9%
	rs2249492	G/A	49.6%	75%
	rs50186619	C/T	24.8%	–
*FGFR2*	rs2981582	T/C	83.7%	78.1%
	rs3135724	G/A	31%	46.9%
	rs2162540	G/A	74.4%	68.75%

Abbreviation: SNPs, single-nucleotide polymorphisms.

The SNPs occur in both classes, except rs50186619. Based on odd ratio analysis ( Table 4 ), if the odd ratio value >1 , it means that tend to happen this SNPs in only one of malocclussion. But if the odd ratio value >1, it means that this SNPs happen in both malocclusion. The SNPs in rs2277632, rs2981582, and rs2162540 affect both classes of malocclusion. However, rs2277632, rs2981582, and rs2162540 are found more in class II than class III. However, rs2249492 and rs3135724 occur more in class III malocclusion than class II. In rs50186619 is specifically found in class II malocclusion ( Table 4 ).

**Table 4:** Odd ratio of each SNPs in class II and class III skeletal malocclusion

Gene	SNPs	Odd ratio		
		Mutation	Class II	Class III
*COL1A1*	rs2277632	0.420 a	0.635	1.511
	rs2249492	3.348 b	1.747	0.522
	rs50186619			
*FGFR2*	rs2981582	0.529 a	0.700	1.324
	rs3135724	1.853 b	1.382	0.746
	rs2162540	0.642 a	0.789	1.23

Abbreviation: SNPs, single-nucleotide polymorphisms.

a < 1 = mutation occurs in both classes.

b > 1= mutation occurs in class II or III.

Factor analysis was performed using the PCA method that is able to explain the variance in the cephalometric indicator data of both class II and class III of skeletal malocclusion. The PC used is one that has an Eigenvalue above 1 and the cumulative variant described has reached at least 50%. In class II malocclusion, a total of Eigenvalue cumulative variance value (59.2%) was formed by PC1 and PC2. Meanwhile, PC1 and PC2 have reached 60.172% of the total cumulative variance in class III malocclusion, so that the PCs used in the next analysis are PC1 and PC2. The highest positive correlation in PC1 in class II is found in Y-axis and the mandibular plane angle, while facial axis has negative correlation same as facial axis. In PC2 in class II, saddle angle shows the highest correlation, where the SNPs of *COL1A1* (rs50186619, rs2277632, and rs2162540) and *FGFR2* (rs2162540) have positive correlation and the SNPs of FGFR2 (rs2981582 and rs3135724) have negative correlation. However, the ratio of posterior cranial base/mandibular height has the highest negative correlation in PC2. Table 3 shows the risk factors of each SNPs are found against the phenotype indicators. rs50186619, which is only found in class II malocclusion samples, makes ANB angle and Y-axis play an important role in PC1, while making facial axis correlates negatively. In rs3135724, ANB has positive correlation in PC1 and ramus position negatively, while articular angle and facial axis do so in rs2162540 ( Table 5 ).

**Table 5:** Summary of PCA

		Class II		Class III	
PC		1	2	1	2
Variance expained a		42.5%	16.7%	44.4%	15.8%
Cumulative variance b			59.2%		60.2%
Variables	rs50186619	Y axisANBFacial axis (-)	Saddle anglePosterior cranial base/mandibular height ratio (-)	–	–
	rs2277632	Y axisFrankfurt mandibular planeFacial angle (-)	Saddle angleArticular angle (-)	Y axisFrankfurt mandibular planeFacial angle (-)	ANBPosterior facial height ratio (-)
	rs2249492	Frankfurt mandibular planeY axisFacial axis (-)	Mandibular maxillary length differencePosterior cranial base/mandibular height ratio (-)	Y axisFrankfurt mandibular planeFacial angle (-)	Upper gonial angleArticular angle (-)
	rs2981582	Y axisFrankfurt mandibular planeFacial angle (-)	Articular angleSaddle angle (-)	Frankfurt mandibular planeLower gonial angleFacial angle (-)	Upper gonial angleArticular angle (-)
	rs3135724	Y axisANBRamus position (-)	Mandibular maxillary length differenceSaddle angle (-)	Lower gonial angleFrankfurt mandibular planeAnterior facial height ratio (-)	Upper gonial angleArticular angle (-)
	rs2162540	Frankfurt mandibular planeArticular angleFacial axis (-)	Saddle anglePosterior cranial base/mandibular height ratio (-)	Frankfurt mandibular planeY axisFacial axis (-)	Saddle angleArticular angle (-)

Abbreviation: PCA, principal component analysis.

a Represents the variance explained by each PC in PCA.

b Shows the cumulative variance explained by each added PC sequentially.

c Displays the variables contributing the most in each PC.

The Javanese class III skeletal malocclusion phenotype is most influenced by lower gonial angle, Y-axis, and the mandibular plane angle in a positive correlation of PC1. On the other hand, facial angle has negative correlation ( Table 5 ). In PC2 of class III malocclusion, articular angle has the highest negative correlation, and upper gonial angle has the most positively correlation.

The presence of a mutation in *COL1A1* rs2249492 in the form of G to A substitution causes class III phenotype to be largely influenced by the positive correlation of Y-axis and the mandibular plane angle, as well as the negative correlation of facial angle. In rs2277632T/C, the ANB angle and posterior facial height also slightly influence the phenotype measurement. In *FGFR2* , rs2981582T/C makes the Frankfurt mandibular plane angle predominantly affect the phenotype, whereas rs2162540G/A makes the saddle angle play an important role in PC2. rs3135724G/A causes the anterior facial height affect PC1 negatively.

## Discussion

This is the first study that report the SNPs of *COL1A1* and *FGFR2* in class II and class III malocclusion in Javanese population. Class II and class III skeletal malocclusion is a polygenic disorder with various environmental risk factors. Although some genetic association analysis studies related to class II and III skeletal malocclusion have been performed, the genetic factors that cause skeletal malocclusion phenotype are still not agreed upon in the general population; so further research is still needed to solve this problem. 10

The human genome is amount of over three billion base pairs, among them 23 paired chromosomes located in the nucleus of each cell. 11 Genes, the basic functional unit of inheritance, are located on each chromosome and code for proteins that are responsible in part, for our phenotypic expression. A phenotype is the outcome morphology based on an individual's genetic code. 12 Every human has two copies (alleles) of every gene, inherited from each parent. Malocclusion is a polygenic trait and because of this it has a complex etiology that is difficult to characterize. 13

Of the three billion base pairs in every human being, a large percentage is identical. SNPs are spots within the genomic sequence where a single base pair set has mutated and differ between individuals. These alleles (alternate forms of the same sequence) can be expressed different characteristic of malocclusion in javanese population. These rare alleles are examined because this study wants to know the effect of SNPs that are selected in class II and class III malocclusion. Mutation in SNPs can occur in promoter genes, exons, and introns. SNPs can greatly affect gene expression through various mechanisms, including changing promoter activity, binding with transcription factors, causing DNA methylation and histone modification, and altering gene transcription and translation. In addition, amino acid substitution caused by the presence of SNPs can change amino acid sequence, secondary protein structure, protein function, and its interactions. 7

The occurrence of class II and III skeletal malocclusion is closely related to abnormal craniofacial morphogenesis, so that it is necessary to carry out further research on various genes related to craniofacial morphogenesis, namely collagen, *FGF* , *FGFR* , bone morphogenesis protein ( *BMP* ), sonic hedgehog, and other factors along the signaling pathway. 14 The *COL1A1* and *FGFR2* genes were selected in this study because their SNPs' odd ratio value was greatest in class III skeletal malocclusion. 8

The *COL1A1* -mediated inhibition of b-catenin allows normal dentoalveolar complex formation. Improper activation in the odontoblasts and osteoblasts can lead to aberrant formation. 15 FGFR families have a reported role in epithelial and mesenchymal interactions in the developing craniofacial complex. FGFR genes are expressed in the developing mandible. 16 Ligands for FGFRs such as FGFs are involved in mandibular outgrowth and morphogenesis. 17 Thus, logic would dictate that the receptors for the FGFs (FGFR) would have a similar effect.

There were five SNPs that were significantly found in both classes in this study, namely rs2277632T/C (65.1% in class II, 46.9% in class III), rs2249492G/A (49.6% in class II, 75% in class III) in *COL1A1* , and rs2981582 T/C (83.7% in class II, 78.1% in class III), rs2162540G/A (74.4% in class II, 68.75% in class III), and rs3135724G/A (31% in class II, 46.9% in class III) in *FGFR2* . Rs50186629C/T is only found in class II malocclusion (24.8%). Based on their odd ratio, rs2249492 more likely occurs in class III malocclusion, while rs3135724 in nonmutated class II malocclusion. In this study, class II malocclusion phenotype was strongly influenced by Y-axis and mandibular plane angle, while class III phenotype was influenced by the lower gonial angle and the mandibular plane angle. These indicators reflect the combination of anteroposterior mandibular position and rotation. Ardani et al support this study that S-Go line has positive correlation with upper anterior dental height and lower posterior dental height) that can cause excessive development of the anterior facial height, then the mandible rotates. 18

The presence of mutations causes changes in the cephalometric indicators that influence the phenotype of class II and III malocclusion the most. For example, ANB angle and Y-axis, which demonstrate relative jaw anteroposterior position, play positive role in rs50186619 and rs3135724 ( Table 5 ) in class II malocclusion; it means that more rs50186619 found in class II will increase the sagittal dimension of maxilla. In rs3135724, less in class II malocclusion than class III means that this SNP has less effect on skeletal growth of mandible than higher risk of class II malocclusion formation.

On the other hand, facial angle in class III malocclusion, which indicates the vertical position of the chin, plays negative role in rs2277632, rs2249492, and rs2162540. In rs2277632 and rs2249492, found in class II and class III, appropriated with the result in Table 3 , that less frequently found rs2277632 will increase facial angle value, which means that higher risk to be class III malocclusion. Otherwise, less frequently found rs2249492 will decrease facial angle value, which means that higher risk is class II malocclusion.

In *FGFR2* , rs2981582T/C makes the Frankfurt mandibular plane angle dominantly positively correlated the phenotype, more frequently found would increase the Frankfurt mandibular plane angle, which means that higher risk to be class II malocclusion, while does so on in rs2162540G/A. rs3135724G/A causes the anterior facial height affect PC1 negatively, more frequently found would decrease the anterior facial height, which means horizontal growth of mandible.

In a Caucasian population with skeletal malocclusion, rs2249492 in *COL1A1* was associated with maxillary and mandibular horizontal discrepancy, whereas rs2981430 in *FGFR2* was associated with facial vertical discrepancy. 8 These genes play an important role in the craniofacial skeletal growth and development. *COL1A1* encodes type I procollagen chain, which is a major structural protein in bone and skin. Mutations in this gene often cause bone abnormalities, like osteogenesis imperfecta. 17 Collagen expression is highly dependent on tissue specificity and its growth and development stage. Most of *COL1A1* expression is not found in adult tissue, unless stimulated by a lesion. 19 Although there is still very limited research on the effect of *COL1A1* SNPs in intron, it has been known that it can increase *COL1A1* expression so that the composition of collagen subunits in the tissue is altered. As a result, the susceptibility to bone fractures is increased in certain population groups. 20 In mice crossed with *Col1a1-cre* , mandibular growth was severely stunted and severe malocclusion occurred. 21

SNPs rs2249492C> T occur in intron whose function is still unknown. However, it may affect gene expression and mRNA stability. 22 The prevalence of SNPs in this region was increased in class III skeletal malocclusion in Caucasian population compared with class I. 8 SNPs at rs2249492 G/A were also found in 70% of patients with Javanese class II skeletal malocclusion. 23 This SNPs has a relationship with the occurrence of pelvic organ relapse, although it is not significant. 24 Diseases related to rs2277632T/C are still unknown.

The FGF signaling pathway is very influential on the primordial facial formation. *FGFR2* can be found extensively on the primordial face, while the FGF ligands are present in certain regions; for example, FGF8, FGF9, and FGF10 are found in the nasal cavity, while FGF2 and FGF4 are found in the frontonasal and mandibular mesenchymal cartilage. Mutations along the FGF signaling pathway can affect craniofacial growth; for example, mutations in *FGFR1* and *FGFR2* can cause craniosynostotic syndromes, such as Apert, Crouzon, and Pfeiffer syndrome, which show mandibular prognathism phenotype. 10

rs2981582 T > C is strongly associated with the occurrence of breast cancer and nonfunctional pituitary adenoma in various races. 25 26 SNPs at rs2981582 change the bond affinity of *FGFR2* to transcription factors (e.g., OCT-1/RUNX2 and C/EBpb) and increase *FGFR2* expression. 27 Rs3135724 is also significantly associated with the occurrence of breast cancer in French population as rs2162540 does so in Caucasian and African-American population. 28 29 In line with a previous study, SNPs at rs2162540 increase the risk of class II and III skeletal malocclusion. 10 However, rs2162540 is only found in another study. Jiang et al mention that craniofacial syndromes caused by FGFR2 mutations often involve multiple early suture fusions, which support this study that higher in class II can be caused by maxillary midline and other facial sutures between the maxilla and zygoma, orbital rim, and nasal bones. 30

All SNPs found in this study occur in introns whose specific function is still unknown. SNP is a genetic variation that occurs in the human genome, which can occur in the coding or noncoding regions of the genome. Some SNPs that occur in exons may not affect gene expression if they produce the same amino acid. SNPs that occur in the promoter region can cause alteration in the promoter activity, its binding characteristics with transcription factors, DNA methylation and histone modification. SNPs in the introns, as in this study, may cause changes in the transcription splicing, protein-binding characteristics, and function of long noncoding RNA or microRNA. It can also affect gene transcription through remote cis effects. 5 Further research is needed to determine the effect of the intron SNPs on the shape, amount, and properties of the protein produced.

However, Jiang et al found that SNPs found in *FGFR2* are all located in intron 2; these SNPs are rs2981578 and rs10736303. These also act as the binding sites to RUNX2 and SMAD4, which increase the expression of *FGFR2* . On the other hand, their minor genotype led to decreased binding affinity, whereas the effects of RUNX2 and SMAD4 works the other way around. RUNX2 and SMAD4 play an important role in osteoblast differentiation and proliferation. 30 31 32

The drawback of this study is the relatively small number of samples because it is quite difficult to obtain samples with skeletal malocclusion who have not undergone any orthodontic treatment in adulthood. The small sample size also led to bias whether the SNPs found were just racial characteristic variations or the indicator of class II or class III skeletal malocclusion. It is advisable to do further research on SNPs on other genes involved in craniofacial bone growth in patients with skeletal malocclusion.

## Conclusion

*COL1A1* , especially rs2249492, may be a risk gene in class III skeletal malocclusion in Javanese population. However, rs3135724 in *FGFR2* occurs more in nonmutated class II malocclusion. rs2981582 occurs the most in both class II and class III skeletal malocclusion, followed by rs2162540 and rs2277632. It is hoped that SNPs can be used as early detection of skeletal malocclusion so that interceptive orthodontic therapy or optimal preparation for orthognathic surgery can be performed to shorten treatment time with more stable results.
